# The moderating role of self‐efficacy in influencing the level of competencies among Middle Nurse Managers: A cross‐sectional study

**DOI:** 10.1111/jnu.70121

**Published:** 2026-08-26

**Authors:** Dhurata Ivziku, Felice Curcio, Lucia Filomeno, Alessio Lachi, Fabio D'Agostino, Noemi Giannetta, Daniela Forte, Nexhmije Gori, Cesar Ivan Aviles Gonzalez, Daniela Tartaglini

**Affiliations:** ^1^ Department of Health Professions Fondazione Policlinico Universitario Campus Bio‐Medico Rome Italy; ^2^ Faculty of Medical Sciences AAB College Pristina Kosovo; ^3^ Faculty of Medicine and Surgery University of Sassari Sassari Italy; ^4^ Department of Clinical and Molecular Medicine Sapienza University of Rome Rome Italy; ^5^ Saint Camillus International University of Health Sciences Rome Italy; ^6^ Department of Biomedicine and Prevention University Tor Vergata Rome Italy; ^7^ Office of Projects and International Relations, Chamber of Nurses, Midwives, and Other Health Professionals Pristina Kosovo; ^8^ University of Enna “Kore” Enna Italy; ^9^ Research Unit of Nursing Science, Department of Medicine and Surgery Campus Bio‐Medico University Rome Italy

**Keywords:** competencies, educational appropriateness, leadership development, Middle Nurse Manager, self‐efficacy, structural equation modeling

## Abstract

**Background:**

Middle Nurse Managers (MNMs) play a pivotal role in translating organizational strategy into operational practice, ensuring care quality, and fostering team performance. Despite their importance, limited research has examined the factors influencing MNMs' competencies, particularly the interplay between individual and organizational determinants.

**Aim:**

To examine the direct and moderating effects of self‐efficacy and other organizational and individual factors on MNMs' competence knowledge and competence application across Italy.

**Methods:**

A cross‐sectional, multicenter study was conducted between September 2022 and December 2023 with the participation of 382 MNMs from public and private healthcare organizations. Data were collected using validated instruments, including the Chase Nurse Manager Competencies Scale, the Questionnaire on Experience and Work Evaluation (QEEW 2.0), and the Leadership Self‐Efficacy Scale. Structural equation modeling (SEM) tested direct effects of educational appropriateness, leadership training intentions, autonomy, role clarity, and work complexity, and the moderating role of self‐efficacy.

**Results:**

Educational appropriateness, autonomy, and role clarity were significantly associated with competence knowledge, while educational appropriateness and autonomy were associated with competence application. Self‐efficacy was directly associated with both competence outcomes and significantly moderated the relationship between educational appropriateness and autonomy with each competence dimension, with positive associations observed among individuals with higher self‐efficacy (*β* = −0.456 for knowledge; *β* = −0.459 for application, *p* < 0.001).

**Conclusions:**

Self‐efficacy was associated with competence outcomes and moderated the associations between organizational factors and competence. High self‐efficacy may reduce reliance on perceived educational appropriateness, whereas lower self‐efficacy increases its importance.

## 1 Introduction

Middle Nurse Managers (MNMs) play a pivotal role in translating organizational strategy into clinical practice by managing resources, coordinating nursing care, and leading teams to achieve high‐quality patient care and organizational performance (González‐García, Pinto‐Carral, Villorejo, et al. 2021). To fulfill these responsibilities, they require competencies in leadership, decision‐making, communication, financial management, and human resource management (Cziraki et al. 2018). As healthcare environments become increasingly complex, developing high levels of managerial competence is essential to ensure quality of care, staff well‐being, and organizational effectiveness (Gutberg and Berta 2017; Warshawsky et al. 2022; Filomeno et al. 2024, 2023).

Nursing management competence is shaped by both individual and organizational factors. Educational appropriateness, continuous professional development, leadership training, and learning intentions support the acquisition of managerial knowledge and skills (Pursio et al. 2024; Aiken et al. 2017; Cummings et al. 2018). Organizational characteristics—including autonomy, role clarity, supportive leadership, and opportunities for professional growth—also influence competence development (Lunden et al. 2017; Aiken et al. 2017; Lee et al. 2019). Additionally, previous leadership and job design research suggest that complex work environments may stimulate learning, adaptive problem‐solving, and competency development by exposing managers to diverse and cognitively demanding situations (Baig et al. 2022; Feng et al. 2025). Therefore, the complexity of managerial work may provide opportunities for learning and professional growth by exposing managers to challenging decision‐making situations. Although greater work complexity may also increase workload and stress, it was hypothesized that exposure to complex managerial tasks would be positively associated with competence development because such experiences provide opportunities to acquire and refine leadership capabilities. Conversely, challenges such as inadequate staffing, high workloads, and limited access to training can hinder competency development. Finally, external determinants, including regulatory frameworks, healthcare policies, and evolving patient care demands, also necessitate nurse managers to continuously adapt and expand their competencies to meet the changing healthcare landscape (Penconek et al. 2021).

Building on these organizational and individual determinants, the nurse manager's belief in their own capabilities—self‐efficacy—plays a central role in shaping and enhancing managerial competencies. Self‐efficacy, defined as the belief in one's ability to accomplish tasks and responsibilities, is a crucial determinant in the development of nurse manager competencies. Managers with high levels of self‐efficacy are more confident in making complex decisions, managing staff effectively, and leading multifaceted organizational processes (Lartey et al. 2023). This confidence enhances motivation for continuous improvement, encouraging engagement in professional development programs and leadership initiatives. Furthermore, successful experiences reinforce self‐efficacy, creating a virtuous cycle whereby increased competence further strengthens confidence in managerial abilities (Cziraki et al. 2018).

Understanding the factors and determinants that influence MNMs' competency levels is essential for developing targeted strategies to enhance leadership effectiveness in healthcare. However, only a few studies in the literature have explored the determinants of nurse managers' levels of competencies (González‐García, Pinto‐Carral, Pérez‐González, et al. 2021).

The present research aimed to examine the determinants of competence knowledge and competence application among Middle Nurse Managers in Italy, with particular attention to the moderating role of self‐efficacy.

Specifically, the study sought to:

1. Assess the associations between selected individual and organizational factors—educational appropriateness, leadership training intentions, autonomy at work, role clarity, and work complexity—and MNMs' competence knowledge and competence application;
2. Examine the direct association between leadership self‐efficacy and MNMs' competence knowledge and competence application;
3. Test whether self‐efficacy moderates the relationships between the selected determinants and MNMs' competence knowledge and competence application.

Based on previous evidence, we hypothesized that educational appropriateness, leadership training intentions, autonomy at work, role clarity, and work complexity would be positively associated with competence knowledge and competence application. We further hypothesized that self‐efficacy would be positively associated with both competence outcomes and would moderate the relationships between the independent variables and competence knowledge and competence application.

## 2 Methods

### 2.1 Design

This study has a cross‐sectional, observational, multi‐center design. To ensure rigorous reporting and transparency in the design and execution of our observational study, the researchers followed the STROBE checklist to promote clarity and reproducibility of our findings (Von Elm et al. 2007).

### 2.2 Theoretical Framework

The present study was guided by the leadership competency framework proposed by Almatrooshi et al. (2016), which integrates transformational and transactional leadership theories and highlights the role of cognitive, social, and emotional competencies in effective leadership and organizational performance. According to this framework, leadership competence is associated with the interaction of individual and organizational factors that support effective managerial behaviors.

In this study, cognitive competencies were operationalized through educational appropriateness and leadership training intentions; social competencies through autonomy at work, role clarity, and work complexity; and emotional competencies through leadership self‐efficacy, defined as managers' confidence in their ability to perform leadership responsibilities effectively. Based on this framework, educational appropriateness, leadership training intentions, autonomy, role clarity, and work complexity were included as independent variables expected to be associated with competence knowledge and competence application, while self‐efficacy was examined as both a direct predictor and a moderator of these relationships.

### 2.3 Sample and Setting

This study presents data gathered from several different healthcare settings, both private and public, throughout the Italian territory. The study was conducted with the support of the Italian Scientific Society for the Direction and Management of Nursing (SIDMI) network. Nurse Managers were invited to complete a set of validated questionnaires between September 2022 and December 2023. The inclusion criteria were as follows: (1) employed in a public or private healthcare organization; (2) have been in their current managerial role for at least one year. All managers fulfilling the inclusion criteria received a Google Forms link to the survey via their institutional email address. Participants were excluded from the study if they were not assigned to a stable work setting. The sampling method for this study was convenience‐based, and participation was voluntary.

Although a convenience sampling strategy was used, several measures were adopted to reduce potential selection bias and enhance sample heterogeneity. Recruitment was conducted across multiple healthcare organizations distributed throughout the Italian territory, including both public and private settings. This multi‐center approach allowed the inclusion of Middle Nurse Managers working in different organizational contexts, geographical areas, and healthcare settings. Moreover, broad inclusion criteria were applied to capture variability in managerial experience, educational background, and workplace characteristics. Nevertheless, because participation was voluntary and based on convenience sampling, the possibility of self‐selection bias cannot be excluded.

### 2.4 Data Collection

Several demographic and professional variables were collected to describe the sample comprehensively. This included age, sex, educational background, tenure, and the region of Italy where participants work.

Educational appropriateness was assessed using two items specifically developed for this study. Responses were rated on a 5‐point Likert scale ranging from 0 (not at all appropriate) to 4 (completely appropriate), yielding a total score ranging from 0 to 8, with higher scores indicating greater perceived educational appropriateness. In the present study, the two‐item scale demonstrated acceptable internal consistency (Cronbach's *α* = 0.745).

Leadership training was assessed using three items designed to measure participants' interest in undertaking leadership training. Responses were rated on a 7‐point Likert scale ranging from 1 (strongly disagree) to 7 (strongly agree), yielding a total score ranging from 3 to 21, with higher scores indicating stronger intentions to participate in leadership training. In the present study, the scale demonstrated good internal consistency (Cronbach's *α* = 0.885).

Three scales were used to identify potential factors contributing to Nurse Managers' competency. The Chase Nurse Manager Competencies Scale (Chase 1994) includes 53 items relating to nurse manager competencies, with each item evaluated in the “knowledge and understanding” and “ability to implement and/or use” sections. Each section's components are divided into five dimensions: technical (items 1–11), human relations (items 12–24), conceptual (items 25–32), leadership (items 33–46), and financial management (items 47–53). The scale uses a 4‐point Likert‐type scoring system for both the “knowledge and understanding” and the “ability to implement and/or use” sections, with each managerial competency rated for its importance as 4 = essential, 3 = contributes significantly, 2 = contributes moderately, and 1 = contributes minimally. The total score for each segment is computed by adding the item scores, and it varies from 53 to 212. The CNMCI was validated in Italian and presented good psychometric qualities (Ivziku et al. 2024). In the present study, the competence knowledge and competence application subscales demonstrated excellent internal consistency, with Cronbach's *α* coefficients of 0.978 and 0.976, respectively.

To measure role clarity (4 items), autonomy at work (4 items), and work complexity (3 items), we used the Questionnaire on Experience and Work Evaluation (QEEW 2.0 SKB) (Van Veldhoven et al. 2015). After twenty years of intensive usage, the QEEW 2.0 has grown into a unique set of brief survey scales that are easily applicable in many practical settings. It has also proven helpful in scientific studies on the functioning of human capital in organizations. The scale uses a 4‐point Likert‐type scoring system from 0 (always) to 3 (never); the score is standardized from 0 to 100, and lower values indicate better results. The scale was previously validated in the Italian population (Pace et al. 2010). In the present study, the role clarity, autonomy at work, and work complexity subscales demonstrated acceptable internal consistency, with Cronbach's *α* coefficients of 0.799, 0.793, and 0.733, respectively.

The Leadership Self‐Efficacy Scale (Hiller 2005) was used to measure self‐efficacy. It is assessed using 7 items, rated on a 7‐point confidence scale ranging from 1 (0% confidence) to 7 (100% confidence). Because no validated Italian version was available, the instrument was translated following forward‐backward translation procedures and subsequently reviewed by a panel of experts before administration. In the present study, the scale demonstrated excellent internal consistency (Cronbach's *α* = 0.959).

### 2.5 Data Analysis

An expected dropout rate of 10% was factored into the calculations to adjust the sample size, accordingly, ensuring robustness against potential data loss. It was estimated that the sample size should be between 310 and 380 participants to achieve 95% power to conduct a multivariable linear regression analysis with a level of significance *p* < 0.05. However, we enrolled 382 participants for a more stable analysis. Sample size was calculated using G*Power 3.1 (Heinrich Heine University). Although the initial sample size calculation was based on the planned regression analyses, the final sample was also considered adequate for structural equation modeling. Consistent with methodological recommendations, sample size adequacy for SEM was evaluated in relation to model complexity and the number of freely estimated parameters rather than a fixed minimum sample size (Wolf et al. 2013). Moreover, the ratio of observations to free parameters exceeded the desirable 20:1 ratio proposed by Jackson (2003) and discussed by Kline (2016), supporting the adequacy of the sample for the hypothesized model. Together with the satisfactory global fit indices, these considerations support the adequacy of the sample for estimating the hypothesized model.

Descriptive statistics were employed to summarize demographic and sample data. Continuous variables were tested for normal distribution and provided as means with standard deviations (SD), whilst ordinal and nominal data were presented as frequencies and percentages.

To investigate the relationships among the collected variables, we employed the Structural Equation Modeling (SEM) approach, as proposed by Hu and Bentler (1999) and Bentler and Yuan (1999). SEM is a well‐established statistical technique for analyzing complex relationships between latent constructs, allowing for the simultaneous estimation of direct, indirect (e.g., mediating), and moderating effects (Byrne 2013).

**FIGURE 1:**
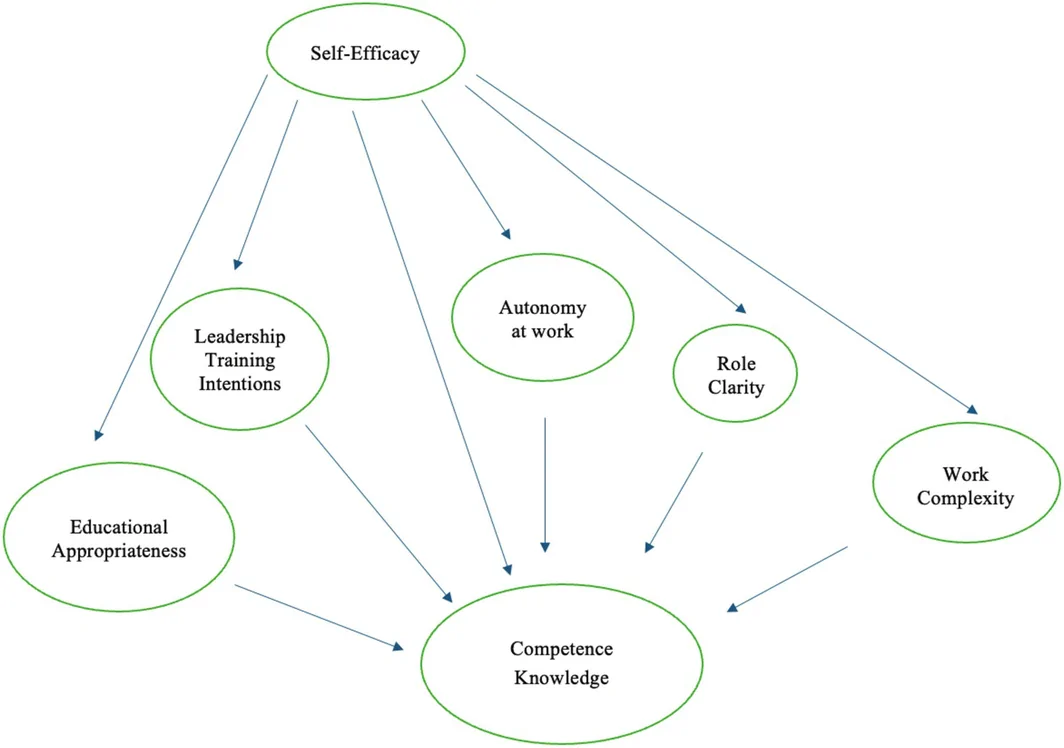
Graphical representation of structural equation modeling of competence knowledge.

**FIGURE 2:**
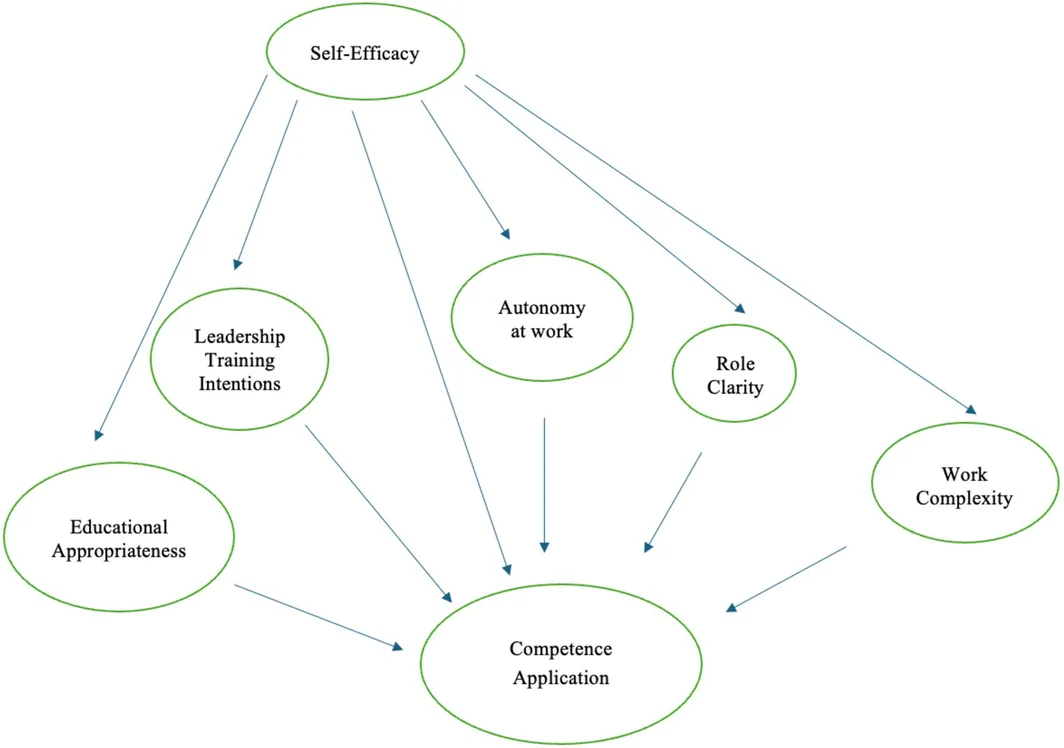
Graphical representation of structural equation modeling of competence application.

Our goal is to investigate the relationship between the dependent construct Competence Knowledge (Figure 1) and Competence Application (Figure 2), with a set of independent variables that include Educational Appropriateness, Leadership Training Intentions, Autonomy at work, Role Clarity, and Work Complexity aspect. Additionally, we aim to test whether the Self‐Efficacy construct acts as a moderating variable in the relationships between the dependent and independent variables.

**FIGURE 3:**
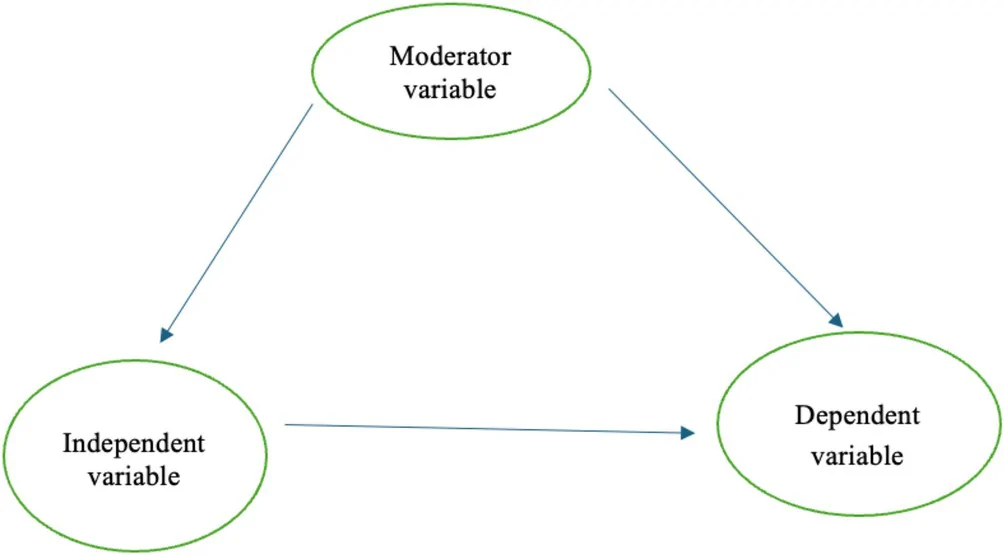
Graphical representation of the moderation model used in the structural equation analyses.

A moderator effect (Figure 3) occurs when the relationship between two variables changes depending on the level of a third variable, called the moderator. The moderator influences the strength or direction of the relationship, indicating that the effect of one variable on another depends on the context or conditions defined by the moderator. This effect is typically tested by including an interaction term between the independent variable and the moderator in a regression model. If the interaction term is statistically significant in explaining the dependent variable, this indicates the presence of a moderation effect.

The goodness of fit values for each model was assessed in accordance with recommendations from the literature (Nunnally 1978; Hu and Bentler 1999). Model fit was evaluated using the Comparative Fit Index (CFI), Tucker‐Lewis Index (TLI), Root Mean Square Error (RMSE), and Standardized Root Mean Square Residual (SRMR). Note that values of CFI and TLI > 0.95, values of RMSEA < 0.06, and values of SRMR < 0.08 indicate good model fit. In addition, standardized regression coefficients (*β*) are presented. Note that all model results of SEM are based on the Maximum Likelihood Estimation method, and for a better model comparison, the reported coefficients are standardized. All the analyses are done with R 4.5.0, an open‐source statistical software.

## 3 Results

A total of 382 completed surveys were received from nurse managers working throughout the entire Italian territory. The dataset was explored for missing data or outliers. Cases with more than 5% missing responses were excluded using listwise deletion. Consequently, the dataset used for SEM contained complete observations for all variables.

The sociodemographic characteristics of the population are represented in Table 1.

**TABLE 1:** Participants' sociodemographic characteristics.

Sociodemographic characteristics	*n* (%)	Mean (range)	± SD
Age		52.14 (30–65)	6.88
Sex			
Female	290 (74.2)		
Male	92 (23.5)		
Working tenure as a nurse in years		24.71 (1–43)	9.58
Working tenure as a manager in years		9.57 (1–35)	9.32
Managed nursing staff		19.46 (1–367)	23.38
Educational qualification			
Two‐year master's degree	43 (11.3)		
First‐level master's degree	291 (76.2)		
PhD	1 (0.3)		
Second‐level master's degree	47 (12.3)		

The descriptive analysis of the variables studied is presented in Table 2. Managers obtained a mean score of 176.41 for the Knowledge and 180.93 for Application sections of the CNMCI suggesting a good level of competencies. The mean scores for Autonomy at work and Role clarity were low suggesting good levels of autonomy and clarity of their roles. Work complexity showed a mean score of 34.19 indicating that MNMs perceive moderate levels of complexity in their roles. Regarding self‐efficacy, results showed a mean score of 30.01, indicating good levels of confidence.

**TABLE 2:** Descriptive analysis of the scores obtained for the individual variables.

	Educational appropriateness	Self‐efficacy	Leadership training intentions	Knowledge score	Application score	Autonomy at work	Role clarity	Work complexity
Mean	5.04	30.01	17.15	176.41	180.93	24.90	17.84	34.19
± SD	1.34	4.49	2.96	24.10	24.16	18.11	14.73	20.11

Managers rated their education as appropriate enough. Lastly, managers' interest in leadership training presented high mean scores suggesting that they were willing and eager to further develop their leadership training.

Competence knowledge and competence application were strongly and positively correlated (*r* = 0.717, *p* < 0.001), indicating that greater perceived knowledge of managerial competencies was associated with greater perceived application of these competencies in practice. This correlation is not sufficiently high to indicate complete overlap between the two competency (knowledge and application) constructs. Therefore, the two dimensions capture closely related but conceptually distinguishable aspects of competence: perceived knowledge of a managerial competency and perceived ability to apply that competency in practice.

Table 3 shows the SEM coefficient of competence knowledge. The model revealed several significant paths, both direct and moderated.

**TABLE 3:** Structural equation modeling of competence knowledge.

	*β*	95% CI		*p*
Direct effects of self‐efficacy				
Self‐efficacy => Educational appropriateness	0.144	0.042	0.246	0.006
Self‐efficacy => Leadership training intentions	0.020	−0.071	0.111	0.672
Self‐efficacy => Autonomy at work	−0.295	−0.396	−0.195	0.000
Self‐efficacy => Role clarity	−0.323	−0.410	−0.236	0.000
Self‐efficacy => Work complexity	−0.240	−0.328	−0.152	0.000
Direct effects of determinants of competence knowledge				
Educational appropriateness => Competence knowledge	0.428	0.246	0.610	0.000
Leadership training intentions => Competence knowledge	0.166	−0.051	0.382	0.134
Autonomy at work => Competence knowledge	0.318	0.111	0.525	0.003
Role clarity => Competence knowledge	−0.260	−0.506	−0.013	0.039
Work complexity => Competence knowledge	−0.102	−0.384	0.181	0.481
Self‐efficacy => Competence knowledge	0.343	0.166	0.521	0.000
Self‐efficacy moderating effects				
Self‐efficacy × Educational appropriateness	−0.456	−0.674	−0.237	0.000
Self‐efficacy × Leadership training intentions	−0.101	−0.398	0.197	0.508
Self‐efficacy × Autonomy at work	−0.298	−0.496	−0.101	0.003
Self‐efficacy × Role clarity	0.170	−0.079	0.418	0.181
Self‐efficacy × Work complexity	0.109	−0.162	0.380	0.430

*Note:* The symbol × indicates the interaction term, and CI the reported confidence intervals are 95%. The CFI = 0.972, TLI = 0.983, RMSE = 0.054, and SRMR = 0.079 indicates an indicative good model fit according to recommended SEM criteria.

First, Self‐Efficacy emerged as a central construct with multiple significant associations. Specifically, it showed positive effects on Educational Appropriateness (*β* = 0.144, *p* = 0.006) and Competence Knowledge (*β* = 0.343, *p* < 0.001), indicating that individuals with higher perceived self‐efficacy tend to perceive a more appropriate education on leadership aspects and also report higher levels of perceived knowledge on the competencies explored. Additionally, Self‐Efficacy was positively associated with Autonomy at work (*β* = −0.295), Role Clarity (*β* = −0.323), and Work Complexity (*β* = −0.240), all with *p* < 0.001 (scales with inverse scores), suggesting that those with higher self‐efficacy tend to perceive better autonomy, role clarity, and more accurate work complexity—possibly reflecting a greater sense of control and mastery. Looking at the determinants of Competence Knowledge, we found that Educational Appropriateness (*β* = 0.428, *p* < 0.001), Autonomy at work (*β* = 0.318, *p* = 0.003), and Role Clarity (*β* = −0.260, *p* = 0.039, inverse score) presented significant positive associations. Leadership training intentions and work Complexity were not significantly associated in this model.

**FIGURE 4:**
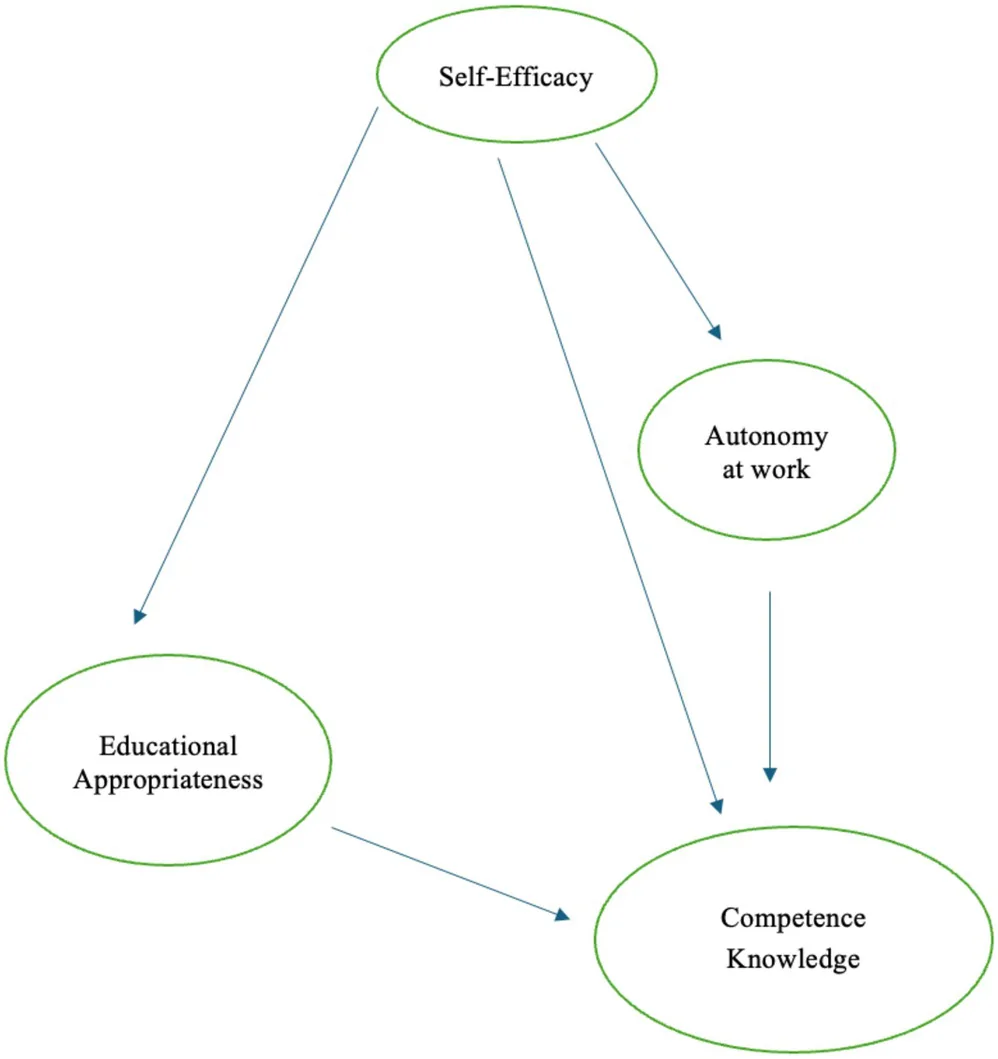
Graphical representation of the moderation effect of self‐efficacy on competence knowledge.

Crucially, we observed a significant moderating effect of Self‐Efficacy (Figure 4) on the relationship between Educational Appropriateness and Competence Knowledge. The interaction term Self‐Efficacy × Educational Appropriateness was negative and significant (*β* = −0.456, *p* < 0.001), indicating a moderation effect. Specifically, the positive association between Educational Appropriateness and Competence Knowledge was weaker among individuals with higher levels of Self‐Efficacy. This finding indicates that the strength of the association between perceived educational appropriateness and competence knowledge varies according to levels of self‐efficacy. One possible interpretation, consistent with self‐efficacy theory, is that individuals with higher self‐efficacy may rely less on perceived educational appropriateness when evaluating their competence. However, because of the cross‐sectional design, this interpretation should be considered tentative and cannot be interpreted as evidence of the underlying psychological mechanism.

This finding is theoretically relevant, as it highlights how personal self‐efficacy beliefs can buffer or reduce the reliance on external factors, such as educational appropriateness, in the development of perceived competence knowledge. It also underscores the importance of considering interaction effects in organizational and psychological models, rather than focusing solely on main effects.

Table 4 shows the SEM coefficient of Competence Application. Also, in this case, the model revealed several significant paths, both direct and moderated.

**TABLE 4:** Structural equation modeling of competence application.

	*β*	95% CI		*p*
Direct effects of self‐efficacy				
Self‐efficacy => Educational appropriateness	0.144	0.042	0.246	0.006
Self‐efficacy => Leadership training intentions	0.020	−0.071	0.111	0.672
Self‐efficacy => Autonomy at work	−0.295	−0.396	−0.195	0.000
Self‐efficacy => Role clarity	−0.323	−0.410	−0.236	0.000
Self‐efficacy => Work complexity	−0.240	−0.328	−0.152	0.000
Direct effects of determinants of competence application				
Educational appropriateness => Competence application	0.441	0.235	0.648	0.000
Leadership training intentions => Competence application	0.113	−0.221	0.447	0.507
Work autonomy => Competence application	0.380	0.117	0.643	0.005
Role clarity => Competence application	−0.161	−0.497	0.175	0.347
Work complexity => Competence application	−0.137	−0.550	0.276	0.517
Self‐efficacy => Competence application	0.325	0.053	0.596	0.019
Self‐efficacy moderating effects				
Self‐efficacy × Educational appropriateness	−0.459	−0.712	−0.206	0.000
Self‐efficacy × Leadership training intentions	−0.024	−0.479	0.431	0.918
Self‐efficacy × Work autonomy	−0.328	−0.578	−0.079	0.010
Self‐efficacy × Role clarity	0.031	−0.298	0.361	0.852
Self‐efficacy × Work complexity	0.124	−0.265	0.514	0.531

*Note:* The symbol × indicates the interaction term, and the reported confidence intervals are 95%. The CFI = 0.971, TLI = 0.968, RMSE = 0.049, and SRMR = 0.079 indicate an indicative good model fit according to recommended SEM criteria.

First, Self‐Efficacy emerged again as a central construct with multiple significant associations. Specifically, it showed positive effects on Educational Appropriateness (*β* = 0.144, *p* = 0.006) and Competence Application (*β* = 0.325, *p* = 0.019), indicating that individuals with higher perceived Self‐efficacy tend to perceive their education as more appropriate and also report greater intention or ability to apply their competencies. Additionally, Self‐Efficacy was negatively associated with work Autonomy (*β* = −0.295), Role Clarity (*β* = −0.323), and Work Complexity (*β* = −0.240), all with *p* < 0.001 (inverse scores), suggesting that those with higher self‐efficacy tend to perceive higher levels of work autonomy, role clarity, and identify better work complexity—again possibly reflecting a greater sense of control and mastery over their work context. Looking at the determinants of Competence Application, we found that Educational Appropriateness (*β* = 0.441, *p* < 0.001) and Work Autonomy (*β* = 0.380, *p* = 0.005) presented significant and positive associations. In contrast, Leadership training intentions, Role Clarity, and Work Complexity were not significantly associated in this model.

**FIGURE 5:**
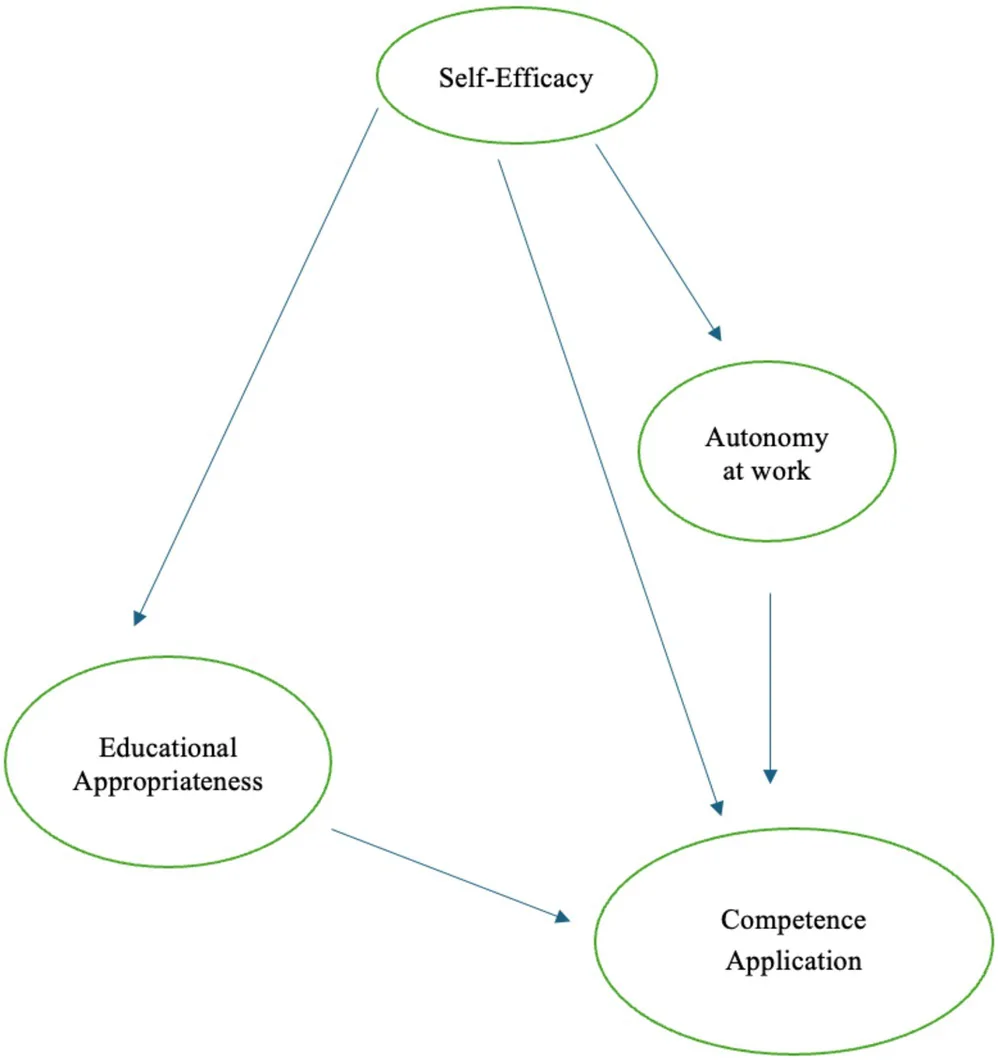
Graphical representation of the moderation effect of self‐efficacy on competence application.

Crucially, we observed a significant moderating effect of Self‐Efficacy (Figure 5) on the relationship between Educational Appropriateness and Competence Application. The interaction term Self‐Efficacy × Educational Appropriateness was negative and significant (*β* = −0.459, *p* < 0.001), indicating a moderation effect. Specifically, the positive effect of Educational Appropriateness on Competence Application was weaker among individuals with higher levels of Self‐Efficacy. In other words, when individuals feel highly efficacious, their perception of educational appropriateness contributes less to their willingness or capacity to apply what they know, suggesting that the positive association between perceived educational appropriateness and competence application was weaker among managers reporting higher self‐efficacy. Although this pattern is consistent with theoretical expectations that self‐efficacy may influence how individuals evaluate or use available resources, the present data do not permit conclusions regarding the psychological processes underlying this association.

This finding further supports the role of personal self‐efficacy beliefs as a protective or enabling resource that can reduce reliance on external environmental factors. It also reinforces the theoretical relevance of including interaction effects when modeling the psychological and organizational predictors of applied behavior.

## 4 Discussion

This study examined the determinants of competencies among Middle Nurse Managers (MNMs) across Italy, focusing on the direct associations of organizational and individual factors and the moderating role of self‐efficacy. Structural equation modeling showed that educational appropriateness, autonomy at work, and role clarity were significantly associated with competence knowledge, whereas educational appropriateness and autonomy at work were associated with competence application. Overall, the findings suggest that MNMs' competencies are associated with the interplay between educational preparation, organizational context, and individual psychological resources.

The positive association between educational appropriateness and both competence outcomes is consistent with previous evidence highlighting the importance of context‐specific education in supporting nurse managers' effectiveness (Djukic et al. 2019; Fischer et al. 2020). In contrast, leadership training intentions were not significantly associated with either competence outcome, suggesting that perceived relevance of educational preparation may be more closely related to managerial competence than a general intention to pursue further leadership training. This finding supports the importance of leadership development initiatives that are practical and closely aligned with the challenges of managerial practice.

Autonomy at work and role clarity were also associated with competence outcomes. Educational appropriateness and autonomy were associated with both competence knowledge and competence application, whereas role clarity was associated only with competence knowledge. These findings are consistent with previous research indicating that clear responsibilities and adequate decision‐making autonomy support nurse managers in coordinating teams, prioritizing tasks, and responding effectively to organizational demands (Cummings et al. 2018).

Autonomy at work was positively associated with competence, knowledge, and its practical application. This finding aligns with prior research suggesting that greater perceived autonomy may support nurse managers in exercising independent judgment, engaging in adaptive problem‐solving, and responding flexibly to the dynamic demands of healthcare environments (Laschinger et al. 2016; Chang et al. 2021; Lommi et al. 2023; Kusakli and Sönmez 2024). By having the freedom to make decisions, nurse managers can implement strategies and initiatives tailored to patient care and team needs, which reinforces their experiential learning and fosters the translation of theoretical knowledge into practical competencies. However, autonomy should not be understood simply as freedom from control. In nursing management, autonomy becomes beneficial when it is accompanied by organizational support, access to resources, and clear expectations. Without these conditions, autonomy may become a source of uncertainty or burden, particularly for managers who feel less confident in their leadership abilities. Therefore, the positive association between autonomy and competence application may reflect the fact that MNMs who perceive greater decision‐making latitude are more able to transform knowledge into concrete leadership behaviors.

Interestingly, work complexity did not emerge as a significant determinant of competence, which is consistent with previous studies indicating that although complex work environments may be motivational and encourage learning, they do not directly lead to measurable competence unless coupled with structured skill development, adequate guidance, and sufficient resources (Van den Broeck et al. 2016; Fischer et al. 2020). This suggests that complexity alone is not sufficient to cultivate managerial proficiency; rather, it must be mediated by supportive organizational structures and targeted educational opportunities. In other words, exposure to complex work situations may create opportunities for learning, but such exposure does not automatically result in competence development. MNMs may require reflective spaces, mentoring, feedback, and adequate organizational resources to transform complex experiences into professional growth. This interpretation is particularly relevant in healthcare settings, where managerial complexity is often associated with workload pressures, staffing challenges, and competing organizational demands.

Self‐efficacy emerged as a central construct, showing direct positive effects on both competence knowledge and competence application, and significant moderation effects on the relationship between educational appropriateness and both competence outcomes. The moderation analyses revealed that the positive associations between educational appropriateness and competence knowledge and competence application were significantly weaker among individuals with higher self‐efficacy. This aligns with Sahebi and Barkhordari‐Sharifabad's (2023) assertion that individuals with strong self‐efficacy rely more on internal mastery expectations than on external validation. Recent evidence supports this buffering mechanism: Shehab et al. (2023) found that knowledge self‐efficacy moderated the relationship between the professional environment and knowledge‐sharing behaviors among head nurses, while Liu et al. (2024) reported that nursing information competence could moderate the relationship between creative self‐efficacy and innovative behavior. These studies illustrate how self‐efficacy can shape the extent to which external conditions influence professional outcomes, paralleling the current study's findings.

The moderating role of self‐efficacy deserves particular attention. The negative interaction between self‐efficacy and educational appropriateness indicates that the positive association between educational appropriateness and both competence knowledge and competence application was stronger among MNMs with lower self‐efficacy and weaker among those with higher self‐efficacy. One possible interpretation, consistent with Bandura's self‐efficacy theory (Bandura 1997), is that managers with lower self‐efficacy may rely more on perceived educational adequacy when evaluating their competence, whereas those with higher self‐efficacy may rely less on this factor. However, given the cross‐sectional design, these interpretations should be considered tentative and do not establish the underlying psychological mechanisms.

Similarly, the moderation involving autonomy suggests that organizational resources may be associated with competence differently according to managers' levels of self‐efficacy. This finding highlights that educational and organizational interventions may not have uniform associations with competence outcomes across all MNMs (Zhao et al. 2019).

The direct association between self‐efficacy and competence outcomes is consistent with recent evidence showing positive relationships between self‐efficacy, professional competence, and work‐related outcomes among nurses (Kallerhult Hermansson et al. 2024). Similar findings have been reported by Liao et al. (2024), who identified self‐efficacy as a mediator between professional identity and learning engagement, and by Basileo et al. (2024), who highlighted the role of self‐efficacy in supporting academic achievement. Although these studies were conducted in different populations, together they support the importance of self‐efficacy as a relevant individual resource in professional development.

Taken together, these findings extend the theoretical framework by Almatrooshi et al. (2016) by showing that leadership competence appears to be associated with the interaction between cognitive, social, and emotional dimensions. Educational appropriateness and leadership training intentions reflect the cognitive dimension of leadership development; autonomy, role clarity, and work complexity reflect the organizational and social context in which leadership is enacted; and self‐efficacy reflects the emotional and self‐regulatory dimension that influences how managers perceive and respond to these conditions. The present findings suggest that self‐efficacy may act as a bridge between contextual resources and competence outcomes, helping to explain why the same educational or organizational conditions may lead to different levels of competence knowledge and competence application among different managers.

The present findings suggest that self‐efficacy is an important individual resource associated with both competence outcomes and the relationships between organizational factors and competence. Consequently, leadership development initiatives should combine context‐specific education with opportunities to strengthen self‐efficacy through practical learning experiences. Structured mentoring, supervised leadership practice, leadership coaching, and multisource (360°) feedback may help managers consolidate managerial skills while aligning perceived and actual competence (Spiva et al. 2020; Sureda et al. 2021; Emam et al. 2024; Shurab et al. 2024).

The moderation findings further suggest that managers may benefit from different development strategies according to their levels of self‐efficacy. For MNMs with lower self‐efficacy, educational programs that provide structured learning, feedback, and guided practice may support competence development. For those with higher self‐efficacy, advanced leadership experiences and reflective feedback may help maintain alignment between perceived and actual competence. Although these implications are consistent with the observed associations, future longitudinal and intervention studies are needed to determine the effectiveness of such tailored approaches.

### 4.1 Practical Implications and Future Directions

These findings have important implications for nursing management practice. Leadership development programs should consider both individual and organizational factors associated with managerial competence. Managers with lower self‐efficacy may benefit from structured education, mentoring, supervised leadership experiences, and constructive feedback, whereas those with higher self‐efficacy may benefit from advanced leadership challenges and reflective feedback to support continued professional development (Shehab et al. 2023).

At the organizational level, healthcare institutions should promote work environments characterized by autonomy, role clarity, and adequate organizational support. Together, these findings suggest that competence development may be strengthened through integrated strategies combining context‐specific leadership education, self‐efficacy development, clear role expectations, and opportunities for continuous professional development.

Healthcare organizations can operationalize self‐efficacy‐building interventions in several practical ways. For example, newly appointed Middle Nurse Managers could participate in structured leadership transition programs that combine mentorship from experienced nurse leaders, supervised participation in complex managerial tasks, and regular reflective debriefing sessions. In addition, organizations could implement periodic leadership coaching and multisource (360‐degree) feedback programs, enabling managers to receive constructive feedback, identify strengths and areas for improvement, and progressively build confidence through guided mastery experiences. These interventions are consistent with Bandura's self‐efficacy theory, which emphasizes mastery experiences, vicarious learning, verbal encouragement, and reflective appraisal as key sources of self‐efficacy development.

Future research should adopt longitudinal and experimental designs to clarify causal pathways among self‐efficacy, educational appropriateness, autonomy, and competence outcomes. Further studies should also examine whether interventions aimed at improving leadership self‐efficacy can enhance competence knowledge and competence application over time. Qualitative and mixed‐methods research may help explain how MNMs interpret autonomy, role clarity, and educational preparation in relation to their confidence and daily leadership practice.

### 4.2 Strengths and Limitations

This study contributes in important ways to research in the field of nursing management competencies. A strength of the study is the inclusion of Middle Nurse Managers from multiple healthcare organizations across different Italian regions and from both public and private settings, which increased the diversity of the sample and allowed the investigation of competencies across heterogeneous organizational contexts. Nevertheless, the present study has some limitations. Because all study variables were measured using self‐report questionnaires completed by the same participants at a single time point, the observed associations may have been influenced by common method bias (or common method variance). Although validated instruments were used and participants completed the survey anonymously, this methodological characteristic may have inflated some of the reported associations. Future studies should consider incorporating multiple data sources, objective performance measures, or longitudinal designs to reduce the potential impact of common method bias. We tried to overcome this by including variability in the number of participants, type of units, hospitals from different regions and public and private settings. Another limitation is the cross‐sectional design of the study, which hinders the identification of cause‐and‐effect relationships and the generalizability of findings.

The use of convenience sampling may have introduced selection bias, as participants who chose to complete the survey may have differed from non‐participants in motivation, leadership development interest, perceived competence, or self‐efficacy. Although recruitment across multiple healthcare organizations and diverse settings increased sample heterogeneity, the findings should be interpreted with caution and cannot be considered fully representative of all Middle Nurse Managers in Italy or generalized to other healthcare systems. Future studies should adopt probability‐based or stratified sampling strategies to improve representativeness and external validity.

## 5 Conclusion

This research highlights the multifaceted determinants of nursing management competencies, emphasizing the interplay of autonomy, role clarity, educational appropriateness, and self‐efficacy. Addressing these factors through targeted interventions and organizational support can enhance nurse managers' effectiveness, leading to improvements in healthcare delivery and work environments. Given the importance of the competencies of Nurse Middle Managers, it is essential to understand the contributing factors. We recommend that future studies on determinants focus on other levels of management and explore additional influencing variables.

## 6 Clinical Resources

American Organization for Nursing Leadership (AONL) Nurse Leader Core Competencies: https://www.aonl.org/resources/nurse‐leader‐competencies.

American Organization for Nursing Leadership (AONL) Certified Nurse Manager and Leader (CNML) Certification: https://www.aonl.org/initiatives/cnml.

American Association of Critical‐Care Nurses (AACN) Nurse Leader Resource and Tool Center: https://www.aacn.org/nurse‐leader‐resources.

American Nurses Association (ANA) Leadership in Nursing Resource: https://www.nursingworld.org/content‐hub/resources/nursing‐leadership/leadership‐in‐nursing/.

World Health Organization (WHO) Nursing and Midwifery Resources: https://www.who.int/teams/health‐workforce/nursing.

STROBE Statement Checklists for Observational Studies: https://www.strobe‐statement.org/checklists/.

## Funding

This study was supported by a grant (reference number 3.24.9) received by the Centro di Eccellenza per la Cultura e la Ricerca Infermieristica (CECRI), Order of Nurses of Rome, Italy. The funding organization had no impact on the design, implementation, or analysis of the research.

## Ethics Statement

The study followed the Declaration of Helsinki's ethical principles and criteria and was approved by the local ethics committee (Number 002.23[40.22] OSS). Before participating in the study, all individuals were given information about the research, the study's aims and voluntary participation requirements. All participants provided online consent before enrolling in the research. The surveys were anonymous, and the results were handled confidentially by the research team. Access to the data was limited to the research team only.

## Conflicts of Interest

The authors declare no conflicts of interest.

## Data Availability

The data that support the findings of this study are available from the corresponding author upon reasonable request.
